# Dietary interventions interact with the perception of effort and enhance endurance performance: a brief narrative review

**DOI:** 10.1080/15502783.2026.2692003

**Published:** 2026-06-24

**Authors:** Barbara Strasser, Johannes Burtscher, Jesus Álvarez-Herms, Martin Kopp, Benjamin Pageaux, Martin Burtscher

**Affiliations:** a Ludwig Boltzmann Institute for Rehabilitation Research, Vienna, Austria; b Faculty of Medicine, Sigmund Freud Private University, Vienna, Austria; c University Hospital for Psychiatry II, Medical University of Innsbruck, Department of Psychiatry, Psychotherapy, Psychosomatics and Medical Psychology, Innsbruck, Austria; d University of the Basque Country (UPV/EHU), Department of Genetics, Physical Anthropology and Animal Physiology, Leioa, Spain; e University of Innsbruck, Department of Sport Science, Innsbruck, Austria; f École de Kinésiologie et des Sciences de l'Activité Physique (EKSAP), Faculté de Médecine, Université́ de Montréal, Montreal, QC, Canada; g Centre Interdisciplinaire de Recherche sur le Cerveau et l'Apprentissage (CIRCA), Montreal, QC, Canada; h Centre de Recherche de l'Institut Universitaire de Gériatrie de Montréal (CRIUGM), Montreal, QC, Canada

**Keywords:** Nutrition, exercise, perceived effort, performance

## Abstract

**Background:**

Exercise tolerance is a pivotal factor in determining athletic competitive success, as well as mobility and quality of life in elderly individuals and those afflicted by chronic ailments. Since tolerance to endurance exercise is closely related to the perception of effort, any measure that influences this perception may also impact endurance performance.

**Methods:**

The aim of this brief review was to evaluate how dietary interventions can improve endurance performance by reducing perceived effort. We contextualize our review within theoretical frameworks that consider effort perception to be a key regulator of endurance performance. Next, we integrate evidence on the ergogenic effects of various dietary interventions with existing knowledge on the perception of effort.

**Results:**

Dietary interventions may enhance endurance performance by improving motor command transmission, slowing fatigue development and related compensatory increase in motor command, and/or modifying the activity of brain networks involved in effort perception and fatigue. Beta-alanine, caffeine or carbohydrate mouth rinsing are examples of evidence-based ergogenic aids. The impact on endurance performance is achieved through their potential to overcome cardiorespiratory and metabolic limitations or through modulation of the central nervous system. Other dietary supplements, such as branched-chain amino acids, citrulline, taurine, and probiotics, may indirectly impact performance by influencing tolerance to physical exertion.

**Conclusion:**

While current evidence supports a key role for dietary interventions on endurance performance by influencing individuals' perceived effort, more research is needed to determine the optimal doses and precise formulations for different sports, in order to employ a personalized strategy.

## 1. Introduction

Individual aerobic endurance capacity determines how long and at what intensity everyday activities, such as climbing stairs or gardening, as well as strenuous sports, including running, cycling, or swimming, can be performed [1]. This capacity is particularly important for athletic competitive success and for maintaining mobility and quality of life in older adults and individuals with chronic illnesses [2,3]. Individual exercise tolerance is defined as the amount of physical exertion that can be sustained before task disengagement and can be increased through appropriate training strategies [4,5]. From a physiological perspective, exercise tolerance is closely related to individual maximum aerobic capacity, that is, the maximal oxygen uptake (VO₂max) [1]. Exercise tolerance may also be represented as the anaerobic threshold, i.e. a certain percentage of the individual VO₂max, that can be maintained for a prolonged time period, e.g. one hour [1].

Exercise tolerance is influenced not only by physiological but also by psychological factors, and both of which influence the perception of effort [6–8]. The perception of effort, also known as perceived exertion, can be assessed using psychophysical scales (e.g. ratings of perceived exertion, RPE scale) during physical exercise, and is frequently used to evaluate the subjective component of exercise tolerance [9,10]. Environmental factors, like heat, cold, or altitude (oxygen deficiency or hypoxia), influence the perception of effort to varying degrees [11]. We recently proposed a psychophysiological framework to explain how hypoxia can influence the perception of effort, and how this perceptual response to exercise could be used as a training tool [12]. We reported higher perceived effort at the same absolute exercise intensity when exercise was performed at high altitude (hypoxia) compared with low altitude (normoxia). In this psychophysiological framework, we hypothesize that repeated exposure to hypoxia may cause adaptations contributing to slowing the development of neuromuscular fatigue, lowering the perceived effort and improving endurance performance [12]. A recent meta-analysis summarized the effects of environmental conditions and exogenous factors, such as nutritional and hydration strategies, on perception of effort and exercise performance [11]. Except for the impact of carbohydrate intake, there is little information on the potential for dietary modification of the perception of effort when exercising and especially when exercising in hypoxic conditions.

The aim of this short review is to outline the current knowledge on how dietary interventions can enhance endurance performance by reducing the perception of effort. First, we position our review within theoretical frameworks that consider effort perception as a key regulator of endurance performance and outline the underlying mechanisms of this perception. We then integrate evidence on the ergogenic effects of various dietary interventions with existing knowledge on perception of effort, focusing on how these interventions may limit the development of fatigue during endurance exercise and thereby influence effort perception. Finally, we propose future research perspectives on dietary interventions and perceived effort.

## 2. Endurance performance and the perception of effort

Human performance is defined as “the measurable output of movement, quantified by metrics such as time, power, distance, or number of repetitions during standardized tasks” [13]. In endurance contexts, these metrics reflect an individual's ability to sustain a given workload over time, to complete a set distance or produce a given power output as quickly as possible, or to maximize distance or power within a fixed duration. Endurance performance is commonly assessed using time-to-exhaustion tests and time trials: the former evaluates the capacity to maintain a constant workload, whereas the latter captures pacing strategies. Both approaches are reliable and valid measures of endurance performance [14,15], and any theoretical model should be able to account for both. Because endurance performance is shaped by physiological [16] and psychological [17] factors, a comprehensive model of its regulation should integrate both domains, and provide sufficient explanatory power to reflect real-world endurance performance (see [13] for details).

### 2.1. Overview of the psychobiological model of endurance performance

Drawing upon the theoretical underpinnings of the intensity of motivation [18], this conceptual framework has been employed within the domain of sports and exercise science for a period exceeding 15 years. It has also paved the way for pioneering studies on the key role of perceived effort in regulating endurance performance [6,19,20]. This model has been developed to explain the regulation of time to exhaustion [19] and time trials [21] and has been shown to be sensitive to physiological (e.g. muscle fatigue or caffeine) [22] and psychological interventions (e.g. mental fatigue or self-talk) [20,23]. For more detailed information on the psychobiological model of endurance performance, the reader is directed to the following publications: [19,24,25]. Briefly, in the context of a time-to-exhaustion test, the psychobiological model of endurance performance predicts that exhaustion, defined as the individual's disengagement from the task, occurs when the individual reaches the maximal effort they are willing to invest. This upper limit is determined by the individual's motivation to succeed in the test. Operationally, exhaustion is proposed to occur when the intensity of perceived effort reaches maximal or near-maximal values on a psychophysical scale. Within this framework, a change in the rate at which perceived effort increases during a time-to-exhaustion test, or a consistently higher or lower perceived effort at a given exercise intensity, should cause the individual to reach their maximal tolerable effort earlier or later, thereby decreasing or increasing exercise tolerance, respectively. In the context of a time trial, the psychobiological model of endurance performance postulates that the conscious regulation of pace is largely governed by the athlete's perceived effort. Consequently, increases in perceived effort—whether induced by muscle fatigue [21] or mental fatigue [26]—or decreases in perceived effort (including same rating for higher power output) through pharmacological modulation [27], lead athletes to adjust their pacing to counteract these negative or positive manipulations. Such adjustments result in impaired time trial performance when perceived effort rises [21], and improved performance when perceived effort is reduced [27]. As perception of effort is a central component of the psychobiological model, and nutritional interventions can improve endurance performance, it is unsurprising that recent meta-analyses have reported reductions in perceived effort following ergogenic nutritional strategies (e.g. [28]. However, although this effect has been observed, the mechanisms by which a nutritional intervention might alter the perception of effort remain to be clarified and warrant further discussion.

### 2.2. Overview of the neurophysiology for perception of effort

Perception of effort is “the conscious sensation of how hard, heavy, and strenuous a physical task is” [7]. It is a distinct perception that reflects both the intensity of the exercise and the individual's degree of engagement in it, and differs from other exercise related perceptions such as pain, temperature or fatigue [7,8]. Perception of effort has been proposed to arise from signals associated with the central motor command [8,29]. A theoretical framework linking effort perception to motor command–related signals is the corollary discharge model of effort perception [8,30]. According to this model, during physical tasks, the perception of effort is generated by the brain's processing of a copy of the central motor command—referred to as the corollary discharge or efference copy. Although the model rules out afferent feedback as a sensory signal of the perception of effort, it considers afferent feedback as an indirect physiological determinant of effort perception [7,8]. The assumption of the corollary discharge model is that sensory feedback contributes to the regulation of the motor command during voluntary actions [29]. In practical terms, any afferent input that modifies motor-command regulation will indirectly influence the perceived effort, without generating the perception itself. For instance, when exercise reduces maximal force‐generating capacity (neuromuscular fatigue and the task remains within the individual's capacity, the individual can compensate [29, 31]. Such compensation can be achieved by increasing the motor command to recruit additional muscle fibers or to counteract inhibition of motor-command transmission to the working muscles. This compensatory increase in motor command amplifies the corresponding corollary discharge and consequently raises the perception of effort [32]. Furthermore, as any perception results from the neuronal process of an internal and/or sensory signal, it is plausible that an alteration in effort perception could also result not only from an alteration in motor command, but also from altered activity in brain areas related to internal state and body awareness, such as the anterior cingulate cortex [33].

Using the corollary discharge model [30], and drawing on the psychophysiological approach we previously introduced to explain how hypoxia interacts with effort perception [12], we hypothesize that nutritional interventions enhancing endurance performance may reduce perceived effort by (i) improving the efficiency of the motor command transmission to the working muscles, and/or (ii) slow the development of neuromuscular fatigue and the associated compensatory increase in motor command, and/or (iii) modifying the functioning of brain regions and networks involved in effort perception and fatigue.

## 3. Nutritional interventions

This section provides examples of dietary interventions that may improve exercise tolerance by reducing an individual's perceived effort during exercise. Beta-alanine, caffeine or carbohydrate mouth rinsing are evidence-based ergogenic aids with a direct impact on exercise tolerance through their potential to overcome physiological limitations or through activation of the central nervous system [34]. Other dietary supplements such as branched-chain amino acids (BCAA), citrulline, taurine, quinine, and probiotics, may indirectly modulate performance via their influence on the tolerance to physical exertion. However, for these supplements, more research is needed before conclusive recommendations can be made regarding their use. Table 1 outlines the main characteristics of selected studies investigating dietary supplementation and exercise-related perception of effort. To better reflect the need for personalization of strategies to both contextual and individual needs, specific conditions (altitude, temperature, humidity, etc.) and their characteristics need to be considered when designing customized dietary strategies.

**Table 1.** Description of selected (more recent) studies investigating dietary supplementation on exercise-related perception of effort (RPE).

Reference	Nutritional aid	Study design	Participants	Intervention	Main findings	Mechanisms
Karayigit et al. [35]	Caffeine	Double-blind, cross-over study	14 M resistance trained Age: 23 ± 2 yrs BM: 83.0 ± 4.0 kg	CMR 250/500/750 mg, 5 s before bench press 1-RM and 60% 1-RM muscular endurance to failure	High-dose CMR ↑ muscular endurance ↓ RPE	Antagonistic interactions with adenosine receptors Increased neurotransmitter release
Page et al. [36]	Taurine	Double-blind, cross-over study	11 M Age: 23 ± 2 yrs BM: 83.0 ± 9.5 kg VO_2_max: 46.0 ± 6.3 ml/kg/min	50 mg/kg BW, 2 h prior to HIEC (cycling at the VT) in the heat (35 °C, 40% relative humidity)	↑ TTE ↑ sweat rate ↓ post-exercise [La] ↓ core temperature and ↓ RPE in the later stages of HIEC	Reduced lactic acid accumulation Lower thermal sensation (reduction in core temperature)
Liu et al. 2025 [53]	Caffeine and Taurine	Double-blind, cross-over study	16 M football players Age: 24 ± 2 yrs BM: 75.0 ± 7.8 kg	Taurine (50 mg/kg)/Caffeine (5 mg/kg)/Taurine (50 mg/kg) + Caffeine (5 mg/kg), 60 min prior to HIEC followed by 6 × 10 sec sprints in hypoxia (simulated 2500 m)	↑ TTE with caffeine and taurine + caffeine ↑ post-exercise [La] with taurine + caffeine ↓ RPE with caffeine	Caffeine = stimulant for the CNS (neural activity, cerebral blood flow, prefrontal cortex), which is linked to cognitive functioning (unaffected by hypoxic stimulation)
Pérez-Piñero et al. 2025 [58]	Beta-alanine	Double-blind, randomized controlled trial	11 M World Tour cyclists Age: 25 (24–28) yrs BM: 69 (67–73) kg VO_2_max: 67.6 ± 1.6 ml/kg/min	20 g per day, 4 daily intakes (breakfast, lunch, snack, dinner) for 1 week	↑ Uphill time-trial (4.5 km, slope of 5%) performance ↑ [La] at the end of the test ↔ RPE	Greater tolerance of sustained anaerobic activity (increased muscle carnosine levels, enhanced intracellular pH buffering capacity)
Luan et al. [37]	BCCA	Double-blind, cross-over study	11 M active students Age: 21 ± 1 yrs BM: 74.8 ± 10.3 kg VO_2_max: 49.0 ± 9.0 ml/kg/min	300 mg/kg per day on consecutive three days prior to HIEC (cycling 1 h at 60% VO_2_max followed by TTE at 80% VO_2_max)	↑ Fat oxidation (during moderate-intensity exercise) ↑ CHO oxidation (during high-intensity exercise) ↑ TTE (trend) and exercise efficiency ↓ VAS (perceived muscle soreness) ↔ RPE	Anti-inflammatory effects Central fatigue-mediated mechanism Enhanced substrate metabolism
Gervasi et al. [38]	BCCA, L-alanine, and CHO	Double-blind, randomized controlled trial	32 (20 M, 12 F) university students Age: 22 ± 1.7 (M), 21 ± 0.9 (F) yrs BM: 68.2 ± 10.9 (M), 52.5 ± 5.3 kg (F) kg	SU (13.2 g CHO, 3.2 g BCAA, and 1.6 g L-alanine), 1 h prior to HIEC (10 × 90 s sprints followed by TTE at 90% VO_2_max) and each training session for 9 weeks	↓ RPE (HIEC) ↓ RPE (REC) ↑ TTE (after 9 week-intake and cycling training)	Improved TRP:BCAA ratio (limiting serotonin synthesis)
Harrington et al. 2023 [61]	BCAA, L-citrulline, and A-GPC	Double-blind, randomized crossover study	30 M trained (≥5 h/wk) Age: 43.7 ± 8.5 yrs BM: 79.2 ± 9.6 kg	SU (8 g BCAAs, 6 g L-citrulline, and 300 mg A-GPC), 7 days prior to HIEC (20 km TTE)	↑ TTE (peak power and time to fatigue) ↔ RPE	NO biosynthesis (increased blood flow, oxygen, and nutrient delivery) Reduced MPB
Schreiber et al [39].	Probiotics	Double-blind, randomized controlled trial	27 M elite cyclists Age: 28.3 ± 5.6 yrs BM: 71.7 ± 7.3 kg VO_2_max: 64.7 ± 5.8 ml/kg/min	Multi-strain probiotic supplement (≈15 billion CFU), daily for 3 months	↔ TTE, VO_2_max ↔ Inflammation (CRP, IL-6, TNF-*α*) ↓ GI symptoms ↓ RPE (TTE test)	Gut-brain axis

Abbreviations: M, male; F, female; BM, body mass; BW, body weight; A-GPC, alpha-glyceryl phosphorylcholine; BCCA, branched-chain amino acids; CFU, colony forming units; CHO, carbohydrates; CMR, caffeine mouth rinsing; CNS, central nervous system; CRP, C-reactive protein; DOMS, delayed onset muscle soreness; GI, gastro-intestinal; HIEC, high-intensity endurance cycling; HRmax, maximum heart rate; IL-6, Interleukin 6; La, lactate; MPB, muscle protein breakdown; NO, nitric oxide; PRS, perceived recovery status; REC, recovery; RPE, ratings of perceived exertion; SU, supplement; TRP, tryptophan; TTE, time to exhaustion; TNF-α, Tumour necrosis factor α; VAS, visual analogue scale; VO_2_max, maximal oxygen uptake; VT, ventilatory threshold; 1-RM, one repetition maximum. Arrow symbols ↑, ↓, and ↔ represent a significant increase, a significant decrease, and no significant change.

### 3.1. Caffeine

It is well established that moderate to high caffeine doses (5–9 mg/kg body mass), ingested before and during exercise, increase endurance performance in laboratory and field settings, but even lower caffeine doses (<3 mg/kg body mass/∼200 mg) can be ergogenic [40]. Caffeine can be ingested or used as a mouth rinse, or as mouth rinsing in conjunction with low-dose caffeine. In the context of ingestion, the ergogenic effects appear to result from antagonistic interactions with adenosine receptors in the central and peripheral nervous systems [41], thereby altering central drive and reducing perception of effort during exercise. Specifically, caffeine has been shown to reduce the amplitude of the motor-related cortical potential measured over motor areas [42], an index of the magnitude of the motor command [32]. Regarding the effects of caffeine mouth rinsing on endurance performance, while these effects remain debated [43], its potential influence has been proposed to be linked to the activation of adenosine receptors in the oral cavity. This activation would lead to increased sympathetic nervous system activity and enhanced brain activation in regions associated with cognitive performance, reward processing, and motor control [44], thereby limiting the negative effects of mental fatigue [44], a phenomenon known to increase perception of effort [45]. Although there is some support of a high-dose of caffeine mouth rinse (750 mg) to increase muscular endurance performance and to decrease perception of effort [35], the majority of the literature has reported no ergogenic effect on aerobic exercise performance [43]. In contrast, the use of all forms of caffeine (chewing gum, bars and gels, drinks) in conjunction with endurance exercise in the heat and at altitude is well supported when dosages range from 3–6 mg/kg and 4–6 mg/kg body mass, respectively [46].

### 3.2. Carbohydrates

Among the variety of dietary strategies employed by athletes to enhance performance, maximizing glycogen stores presents a key strategy. Elevated dietary carbohydrate intake before exercise (glycogen loading) increases both muscle and liver glycogen availability and improves endurance exercise capacity and performance, particularly in events lasting between 60 and 90 min [47]. Maintaining blood glucose levels close to pre-exercise levels through carbohydrate consumption during exercise helps sustain carbohydrate oxidation, improves muscle energy balance and reduces liver glycogen breakdown, and delays the development of fatigue. While glucose ingestion during exercise has minimal effects on net muscle glycogen utilization, it increases muscle glucose uptake and decreases liver glucose output [48]. Notably, while the consumption of carbohydrates can delay the onset of fatigue, it does not prevent it from occurring [47]. Furthermore, rinsing the mouth with carbohydrates can enhance performance by stimulating oral receptors that are connected to brain regions responsible for motor control and motivation [49]. Overall, carbohydrates can significantly contribute to slowing the development of neuromuscular fatigue [50], which in turn will slow the associated compensatory increase in motor command, thereby attenuating the concomitant increase in perception of effort.

### 3.3. Taurine

Taurine is a highly abundant amino acid in skeletal muscle and plays a role in many cellular functions. Taurine dosing appears to be effective at 1–3 g/day, when administered over a period of 6–15 days (1–3 h before exercise), and may improve aerobic and anaerobic performance, recovery, by reducing delayed onset muscle soreness and metabolism, as indicated by markers such as inorganic phosphate and lactate [51]. A double-blind, randomized crossover study in a group of 11 healthy males provides evidence for a role of taurine in thermoregulatory processes. Taurine supplementation (50 mg/kg body mass) administered 2 h prior to exercise in the heat (35 °C) increased time to exhaustion by 10% and sweat rate by 12.7%, while decreasing effort perception and core temperature in the later stages of exercise by 10%, as well as post-exercise blood lactate concentration by 16.5% [36].

Based on a current network meta-analysis examining the effects of different nutritional supplements on endurance performance and athletes' perception under high-temperature conditions, taurine supplements seem to be effective in improving subjective thermal comfort and endurance performance [52]. A recent study investigated the effects of taurine combined with caffeine, administered 60 min before exercise, on endurance performance and subsequent repetitive sprint performance and perception of effort under hypoxic conditions (simulated at 2500 m) [53]. In 16 male university football players, both caffeine (5 mg/kg) and taurine co-ingestion (50 mg/kg) significantly improved endurance performance, whereas neither intervention affected subsequent repetitive sprint performance.

Considering the current state in the literature, taurine may alleviate neuromuscular fatigue by reducing lactic acid and inorganic phosphate accumulation and by enhancing antioxidant capacity. Owing to its positive effect on neuromuscular fatigue and cognition, taurine is therefore likely to slow the fatigue-induced compensatory increase in motor command and to act positively at the cerebral level, leading to decreased effort perception.

### 3.4. Menthol

The application of menthol topically and the use of mouth rinses seem to be ergogenic, especially in hot environments. Both application forms may improve endurance performance and perception, albeit with differing effects on body temperature regulation [54]. The application of menthol to the skin may be advantageous (by lowering thermal sensation) for activities where heat tolerance is a restricting factor. However, due to the cooling sensation it may also increase the risk of heat-related illness [54]. The performance-enhancing effects of menthol mouth rinsing are thought to be linked to its thermal, ventilatory, analgesic and arousing properties [55]. Taken together, menthol is a relatively new option that can benefit endurance performance, particularly by influencing the perception of effort.

### 3.5. Beta-alanine

Beta-alanine supplementation of ∼3–6 g per day is proposed as a novel ergogenic aid for high-intensity exercise performance due to its pH buffering capacity in skeletal muscle which may serve to lower perception of effort [56]. Consequently, this may allow for longer exercise sessions and enhanced training adaptations. One study evaluated the acute effects of ingesting 1.6 g of beta-alanine 30 min prior to exercise on various performance parameters in female cyclists. Although there were no significant changes in mean power or peak work, beta-alanine resulted in a lower RPE compared to the placebo [57]. A recent study supports the effectiveness of one-week high-dose of beta-alanine supplementation (4 × 5 g per day; 155 total cumulative amount) in World Tour cyclists to improve their uphill time-trial performance (4.5 km with a slope of 5%; requiring substantial contribution from anaerobic metabolism) and to minimize fatigue associated with a cycling training camp [58]. This ergogenic aid could be particularly relevant in periods of high training loads, when fatigue levels increase in parallel to training intensity [58]. A systematic risk assessment found no significant health risks associated with the usual dosage of beta-alanine supplements [59].

### 3.6. Branched-chain amino acids

Branched-chain amino acids (BCAA), which include leucine, isoleucine, and valine, are essential nutrients that play a key role in muscle metabolism. Supplementary BCAA may help to reduce muscle damage and fatigue that is associated with endurance exercise [60]. A recent double-blind, randomized crossover study in 11 healthy males supports positive effects of BCAA on substrate metabolism and post-exercise fatigue following endurance exercise [37]. BCAA supplementation (300 mg/kg per day) for three consecutive days prior to exercise stimulated fat oxidation during constant load exercise at 60% VO_2_max. It enhanced carbohydrate oxidation and exercise efficiency during subsequent high-intensity exercise, and reduced immediate post-exercise fatigue compared with placebo [37]. These results indicate the critical role of BCAA in regulating cellular processes to meet the high metabolic demands of exercise and in modulating central nervous system function, thereby counteracting fatigue.

BCAA are also included in the compositions of dietary supplements, where it is difficult to determine their individual effects on exercise tolerance. For example, in a randomized double-blind placebo-controlled trial in 32 healthy subjects, the consumption of a sports nutritional supplement containing BCAA (3.2 g), L-alanine (1.6 g), and carbohydrates (13.2 g) reduced the perception of effort during high-intensity endurance cycling tests and improved time to exhaustion over a 9-weeks supplementation period [38]. Moreover, the reduced perception of effort allowed participants to sustain higher workloads. Another randomized, double-blind, crossover study in 30 trained male cyclists suggests the combination of BCAA, L-citrulline, alpha-glycerylphosphorylcholine (A-GPC) supplementation containing 8 g BCAA, 6 g L-citrulline, and 300 mg A-GPC, may represent an ergogenic dietary approach, particularly for cycling and other sports requiring high levels of muscular strength and endurance [61]. However, the findings of a recent systematic review do not suggest that BCAA supplementation meaningfully improves endurance performance or muscle recovery [62].

### 3.7. Probiotics

Probiotics may benefit athletes indirectly by maintaining gastrointestinal function and health which prevents the immunosuppressive effects and respiratory tract infections caused by intense exercise [63,64]. Furthermore, multi-species probiotic supplementation over a 3 to 4-month period was associated with higher training loads versus placebo and lower perception of effort during a time to exhaustion test [39] and the Wingate test [65], confirming the potential use for performance enhancement in athletes. A few double-blind, randomized, placebo controlled studies on probiotics and athletic performance have demonstrated positive effects of supplementation on endurance performance (*Lactobacillus plantarum* PS128) [66] and the potential to reduce exercise-induced muscle damage and to improve recovery following intense exercise (*Bacillus coagulans* GBI-30, 6086) [67], which was evident not only in the prevention of systemic endotoxemia and inflammation when exercising in the heat (via a combination of *Lactobacillus, Bifidobacterium, and Streptococcus* strains) [68], but also in the reduction of perceived fatigue [69]. Likewise, findings from a recent systematic review suggest that probiotics, specifically a multi-strained probiotic at a minimum dosage of 15 billion colony-forming units daily for a duration of at least 28 days, may contribute to the reduction of fatigue [70], possibly via a microbiome-dependent gut–brain pathway [71].

Previously, Álvarez-Herms et al [72]. had put forward a hypothesis regarding the possible influence of the gut microbiota on the perception of exertion, fatigue, and exercise tolerance. The authors described how the gut microbiota has a potential impact on the brain via the vagus nerve by activating the brain signaling. Consequently, afferent signals originating in the gut promote cerebral sensations and perceptions. In fact, gut-brain communication constitutes a dynamic system that responds to physical demands with specialized microbiota producing metabolites such as brain-derived neurotrophic factor, serotonin, dopamine, GABA, butyrate, and others to communicate with the brain or the central nervous system [73,74].

Short chain fatty acids (SCFAs), particularly butyrate, produced from the fermentation of dietary fiber, may modulate substrate metabolism in skeletal muscle and influence the barrier and immune functions of the intestinal epithelium [75,76]. Furthermore, meta-omic analysis of elite athletes revealed that lactate generated during sustained bouts of exercise could be accessible to the microbiome and converted to these SCFAs (predominantly propionate) that improved endurance performance [77]. The results of many studies confirm the beneficial effect of probiotics on the production of SCFAs by gut microbes [78], which may influence tolerance to physical exertion over a longer timeframe through repopulation and adaption of the gut. In addition, microbiome-dependent production of endocannabinoid metabolites in the gut stimulates the transmission of gut-derived signals to the brain, which may enhance the motivation for exercise via the gut-brain pathway, suggesting a possible mechanistic basis for understanding interindividual variability in exercise motivation and performance [71]. However, the appropriate dosages of probiotics for athletes require further analysis and studies that consider not only the state of athletes' gut microbiota but also investigate the pathophysiological relationship between exhaustive exercise and gut-brain axis as recent studies found detrimental effects of high-intensity training on gut microbiome composition, gut barrier function and crosstalk between gut and blood-brain barrier, which may determine worsening of cognitive performance and mood state [79,80].

**Figure 1.**
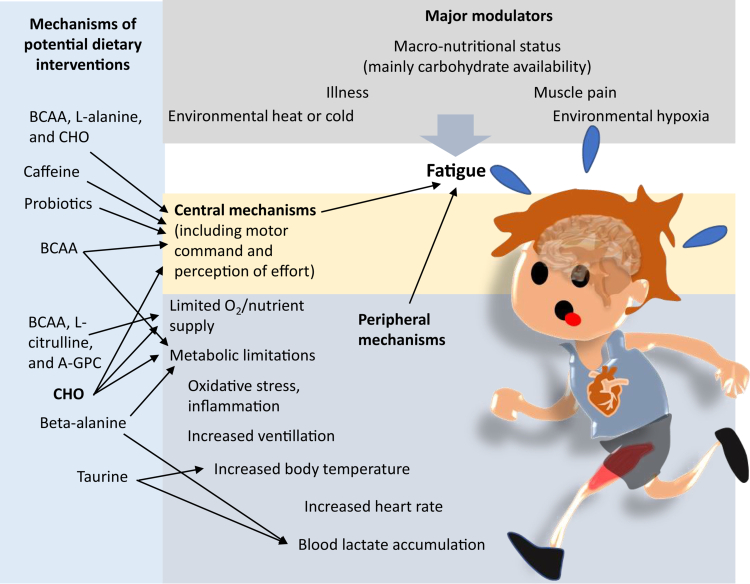
Mechanisms by which primarily the physiological component (muscle energetics) of perceived effort is influenced.

A summary of the potential mechanistic effects of the discussed nutritional strategies on fatigue is provided in Figure 1.

## 4. Perspectives and conclusion

Several dietary interventions may improve athletic performance by modulating the perception of effort, yet many open questions remain. These include factors contributing to ergogenic efficacy, such as inter-individual variability (e.g. genetics, sex, training status) and dosing protocols. With regard to dosing, aspects like absolute versus relative dosing, co-ingestion with other nutrients, and timing and frequency (e.g. acute administration before or during exercise versus chronic protocols) are likely to induce differential effects and must be considered in both study design and results interpretation. Current evidence supports the use of ergogenic dietary supplementation, particularly for endurance performance. However, designing optimally customized protocols for dietary interventions is challenging and requires further research to determine optimal doses and precise across sports, moving towards personalized nutrition. In this context, there is an urgent need for research in the following important areas:

(i) Identification of biomarkers to distinguish specific ergogenic effects (e.g. anti-fatigue, reduced muscle pain, or enhanced energy), leveraging large datasets (e.g. OMICS) and machine-learning/artificial intelligence approaches to disentangle the notoriously complex mechanisms of dietary interventions;
(ii) Randomized controlled trials on elite athletes across sports with differing physiological and cognitive demands to identify discipline-specific vulnerabilities of specific sports disciplines. Such studies may also be of interest for general and clinical populations, who may benefit from improved exercise tolerance to enhance engagement in and adherence to regular physical activity;
(iii) The design of precision formulations that may offer more effective outcomes than generic supplements. This includes optimization of pharmacokinetics and pharmacodynamics and may refer to the route of administration, safer or more efficient administration modalities, prolonged duration of (pharmacological) activity, and organ-specific targeting. Moreover, the identification of evidence-based synergistic supplement combinations, consisting for example of multiple compounds with complementary effects on fatigue (see Figure 1), are promising avenues for future research, as well as for athletic and commercial applications.

To conclude, current evidence suggests that dietary interventions can enhance endurance performance by modulating the perception of effort through both physiological and central mechanisms. Future research should move beyond generic supplementation strategies and instead target the mechanisms that shape the perception of effort to develop more precise and effective nutritional interventions for athletes.

## Data Availability

All data are presented in the manuscript.
